# Polystyrene-bound thioxanthone – a heterogeneous photocatalyst for alcohol oxidation *via* singlet oxygen production

**DOI:** 10.1039/d5cy01464f

**Published:** 2026-02-02

**Authors:** Max Schmallegger, Luis Herbst, Renata Raptová, Mathias Wiech, Dana Dvoranová, Dmytro Neshchadin, Michal Zalibera

**Affiliations:** a Institute of Physical and Theoretical Chemistry, Graz University of Technology Stremayrgasse 9 8010 Graz Austria schmallegger@tugraz.at; b TH-Köln; Fakultät 11, Campus Leverkusen Campusplatz 1 51379 Leverkusen Germany; c Institute of Physical Chemistry and Chemical Physics, Slovak University of Technology in Bratislava Radlinského 9 SK-81237 Bratislava Slovak Republic

## Abstract

We have synthesized a photocatalyst in which thioxanthone is covalently attached on a polystyrene (**TX@PS**) polymer and investigated its activity in the photocatalytic oxidation of alcohols to aldehydes. Upon LED irradiation (405 nm), benzyl alcohol is converted to benzaldehyde at a yield of 73% and hydroxymethylfurfural forms formic acid at 99%. Employing the photocatalyst for consecutive runs (up to three) results in product yields of 51% for the oxidation of benzaldehyde and 98% for hydroxymethylfurfural. EPR provides evidence that the oxidation proceeds *via* a singlet oxygen route. This proof-of-concept study shows that **TX@PS** can straightforwardly be prepared. Using this catalyst provides yields reflecting comparable systems and allows a convenient isolation of the products.

## Abstract 2

A heterogeneous photocatalyst with thioxanthone moieties bound to polystyrene is used for alcohol oxidation *via* singlet oxygen under LED irradiation.

## Graphical abstract

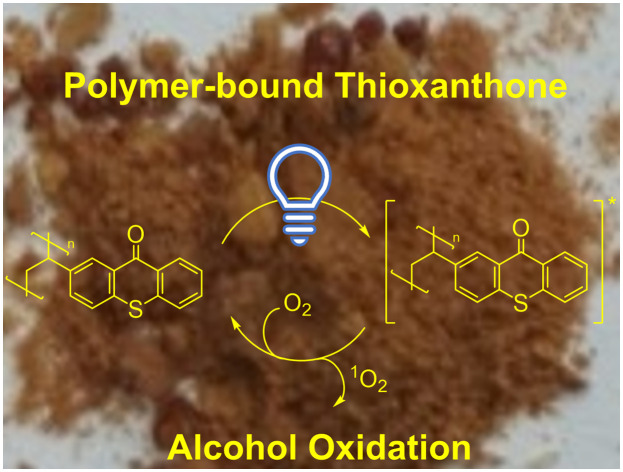

## Introduction

Photocatalysis has emerged as a pivotal technology for addressing global environmental and energy challenges. By utilizing light energy to drive chemical reactions, photocatalysis offers a sustainable and efficient pathway for applications such as water purification, air detoxification, hydrogen production, and CO_2_ reduction.^1–4^ Unlike conventional catalytic processes that necessitate high temperatures and pressures, photocatalysis operates under mild conditions, thereby reducing energy consumption and environmental impact.

In heterogeneous catalysis, a solid photocatalyst facilitates reactions in liquid or gaseous media. This technique offers several advantages, including facile catalyst recovery, high stability, and suitability for large-scale applications.^5,6^

Despite its advantages, photocatalysis faces challenges such as low quantum efficiency, rapid charge recombination, and limited visible light absorption. The development of novel photocatalysts with enhanced stability, efficiency, and selectivity is critical for expanding the practical applications of this technology.^7–9^

This study presents an approach that uses polymer-bound thioxanthone (**TX**) as a photosensitizer for heterogeneous photocatalysis. Thioxanthone functions as an effective electron transfer (ET) agent and triplet energy (EnT) photosensitizer.^10–13^ It has been employed as a photocatalyst in cycloadditions, photoinduced rearrangements, alkene isomerization, and other transformations.^13–15^ In many cases, **TX** has been investigated in bulk solutions, but **TX** moieties have also been incorporated into supramolecular structures such as metal–organic frameworks (MOFs), covalent organic frameworks (COFs), covalent organic cages (COCs), metal–organic cages (MOCs), and (porous) polymers (PPs). The reactivity of organic molecules in confined spaces differs from that in isotropic solutions. For example, ground-state reactions within the cavities of supramolecular systems, like MOFs, COFs, COCs, MOCs, and PPs, have shown accelerated reaction rates, enhanced regioselectivity and stereoselectivity, and in some cases, different reaction mechanisms than in the bulk solution.^16–21^

**Scheme 1:**
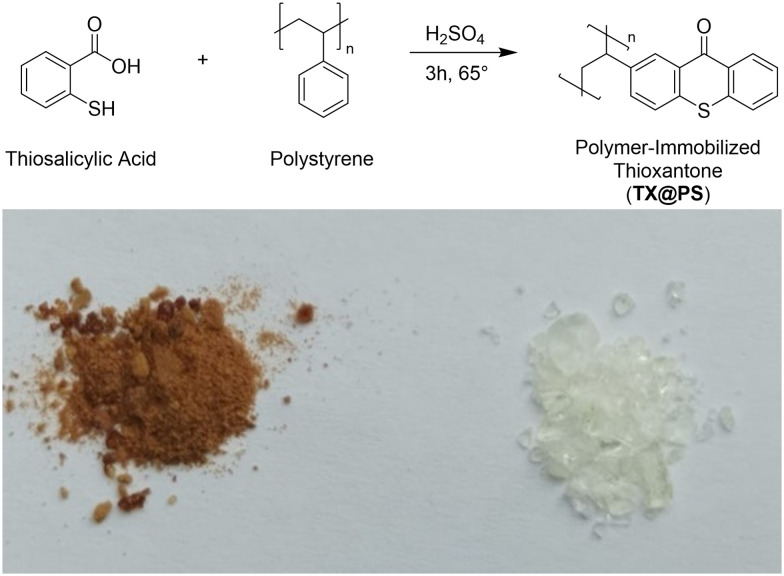
(Top) *In situ* formation of **TX** moieties by reaction of polystyrene and thiosalicylic acid in the presence of sulfuric acid (H_2_SO_4_), yielding **TX@PS**; (bottom) photograph of **TX@PS** (left) and polystyrene (right).

Polymers offer several advantages as a platform for photocatalysis: they can be synthesized from a range of monomers with different properties and varying pore sizes, providing flexibility to fine-tune interactions with guest-molecules.^22,23^ Such polymers can be examined in the solid state or when dissolved, allowing for reactivity and analysis in both liquid and solid states. To study **TX** incorporated into (porous) polymers, this research utilizes the possibility of directly grafting **TX** moieties onto polystyrene *via* an *in situ* process (Scheme 1). The photosensitizing moiety thus becomes covalently attached to the polymer backbone (**TX@PS**).^10,24,25^

This approach produces a polymer with distinct photochemical properties and provides precise control over the photocatalytic oxidation of alcohols under mild conditions. The strategic placement of **TX** within the polymer matrix ensures stability and minimizes the risk of removal by solvents, thereby enhancing the overall efficacy of the system.

## Experimental section

### Materials

All chemicals employed in this study were purchased from commercial suppliers and used without further purification.

### Preparation of polymer-bound thioxanthone

Polymer-bound thioxanthone was prepared using a two-step procedure. First, we prepared polystyrene using a photochemical approach: 170 mg of the bisacylphosphane oxide photoinitiator Irgacure 819 (BAPO) were dissolved in 5 ml of methanol and mixed with 5 ml of a styrene/divinylbenzene crosslinker mixture (volume ratios 2.8 mL/2.2 mL, 3.3 mL/1.7 mL, 3.8 mL/1.2 mL and 4.3 mL/0.7 mL, respectively). The solutions were irradiated at 405 nm for one hour. The obtained polymers were washed with ethanol and dried overnight at 60 °C.

In the second step, thioxanthone moieties were synthesized on the backbone of polystyrene *via* a reaction with thiosalicylic acid (Scheme 1). For this, 1 g of the polymer was added to a 20 mM solution (2 ml) of thiosalicylic acid in concentrated sulfuric acid. The mixture was stirred at 65 °C for 3 h. The resulting polymer was washed with ethanol and dried at 60 °C overnight.

### Characterization of the polymer-bound thioxanthone catalyst

#### UV-vis spectroscopy

UV-vis spectra were recorded on a fiber-coupled diode array photo-spectrometer (Tidas S500, J&M Analytik AG) in conventional quartz cuvettes.

#### ^1^H nuclear magnetic resonance (NMR) spectroscopy

^1^H NMR spectra were recorded on a 400 MHz Bruker Avance III spectrometer. Chemical shifts (*δ*) are reported in ppm relative to tetramethylsilane (TMS), using the residual non-deuterated solvent as an internal reference. Reaction yields were determined directly from the ^1^H NMR spectra by comparing the integrals of the characteristic signals of the product and educt (see SI).

#### Fourier-transform infrared (FT-IR) spectroscopy

FTIR spectra were obtained using a Bruker ALPHA spectrometer. For all measurements, 32 scans were recorded over the range of 4000 cm^−1^ to 400 cm^−1^. The as-synthesized (modified) polymers were measured as dried powder directly in ATR mode.

#### Gas sorption (BET)

The specific surface area (SSA) of the materials was measured *via* nitrogen adsorption measurements using the Brunauer–Emmett–Teller (BET) adsorption theory. Measurements were carried out on a TriStar II 3020 system (Micromeritics, USA), using a 5-point analysis at nitrogen partial pressures (*p*/p*) ranging from 0.06 to 0.20. Prior to the measurements, the samples were degassed under vacuum (VacPrep 061, Micromeritics, USA) for 2 h at 100 ± 2 °C.

### Evaluation of singlet oxygen production by electron paramagnetic resonance (EPR) spectroscopy

EPR experiments were conducted using a Miniscope MS300 X-band EPR spectrometer (Magnettech). An aqueous solution of the sterically hindered amine 4-oxo-2,2,6,6-tetramethylpiperidine (TEMP; 50 mM) and 10 mg of the **TX@PS** photocatalyst was irradiated with a high-power LED (*λ*_max_ = 405 nm) under an ambient O_2_ atmosphere for 30 min.

### Polystyrene-bound TX as a heterogeneous photocatalyst for alcohol oxidation

10 mg of the **TX@PS** photocatalyst were added to a 10 mM solution (benzyl alcohol in CH_3_CN-d_3_ or 5-HMF in H_2_O-d_2_, respectively; volume = 1 mL). The solution was bubbled with O_2_ for 10 minutes and irradiated with a high-power LED (*λ*_max_ = 405 nm) for 18 h. Afterwards, the catalyst was separated from the reaction mixture by centrifugation, washed with ethanol and dried at 60 °C overnight for subsequent use. The reaction mixture was directly used for product analysis with ^1^H NMR.

## Results and discussion

### Photochemical formation of polystyrene

#### Immobilization and characterization of **TX** moieties on the polystyrene backbone

**Fig. 1:**
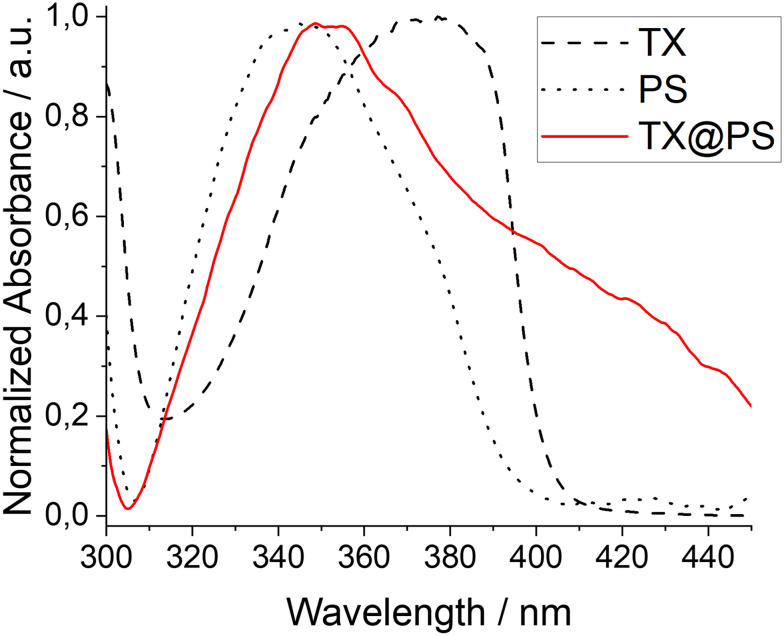
Normalized optical spectra of solid polystyrene (black, dotted line), polystyrene-immobilized thioxanthone (red, solid line) and **TX** in acetonitrile solution (black, dashed line).

Polymer-bound thioxanthone was prepared in a two-step procedure. First, we prepared polystyrene *via* photoinduced radical polymerization (see Experimental section for details). In the second step, thioxanthone moieties were synthesized on the polystyrene backbone *via* a reaction with thiosalicylic acid, as shown in Scheme 1. The formation of thioxanthone is rationalized by the distinct colour change observed after synthesis. UV-vis spectroscopy confirms the formation of a new chromophore on the PS backbone (Fig. 1). Furthermore, IR spectroscopy reveals new vibrational bands in **TX@PS** compared to **PS**. A comparison with commercial **TX** rationalizes that these new bands correspond to the formation of **TX** on the polymer backbone. The corresponding spectra are presented in the SI (Fig. S1). When the polymer is heated in sulfuric acid in the absence of thiosalicylic acid, a greyish powder is obtained, substantiating that the colour change is due to the formation of the thioxanthone chromophore on the polystyrene backbone (SI, Scheme S1).

For the reactants to access the catalytically active species efficiently, it is critical that the composite provides a sufficiently high surface area. BET measurements revealed that **TX@PS** is mesoporous, with a surface area of approximately 5.4 m^2^ g^−1^ (see SI, Fig. S10).

### Chemical stability of **TX@PS**

The chemical stability of heterogeneous photocatalysts is critical to ensure catalytic activity over prolonged periods and avoid impurities in the final product. To evaluate the chemical stability of the **TX@PS** system and its potential application under different solvent conditions, it was dispersed under three distinct solute conditions: nonpolar aprotic (toluene), polar aprotic (acetonitrile), and polar protic (water) conditions. NMR spectra were recorded for the solutions after storing them at room temperature and 70 °C for 24 h. No signals attributable to dissolved **TX@PS** are detected in water and acetonitrile, whereas clear signals corresponding to the dissolved polymer are detected in toluene (see SI, Fig. S4). While crosslinked polystyrene (PS) is generally insoluble in toluene,^26^ the detection of dissolved fractions indicates the presence of non-crosslinked or lightly crosslinked polystyrene chains, showing the potential limitations of this approach for application in nonpolar aprotic solvents, while still rationalizing its use in the other two solvent systems.

### Polystyrene-bound **TX** for photochemical singlet oxygen production

**Scheme 2:**
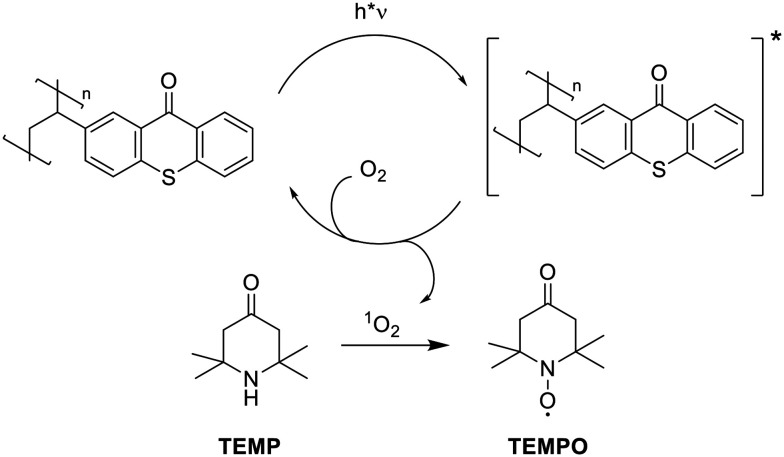
Singlet oxygen production *via* energy transfer from photoexcited polystyrene-bound **TX** and subsequent oxidation of TEMP, resulting in the formation of the persistent TEMPO radical.

The ability of the polystyrene-bound thioxanthone to produce singlet oxygen was evaluated using electron paramagnetic resonance (EPR) spectroscopy. The photocatalyst was dispersed in an oxygen-saturated aqueous solution of the EPR-sterically hindered amine TEMP. Upon irradiation, the polystyrene-bound moieties undergo excitation and intersystem crossing to the triplet state. Subsequently, this excited triplet state undergoes energy transfer to molecular oxygen, generating singlet oxygen.^13^ This singlet oxygen further reacts with TEMP to form a persistent TEMPO radical, which can be conveniently detected by EPR (Scheme 2).

**Fig. 2:**
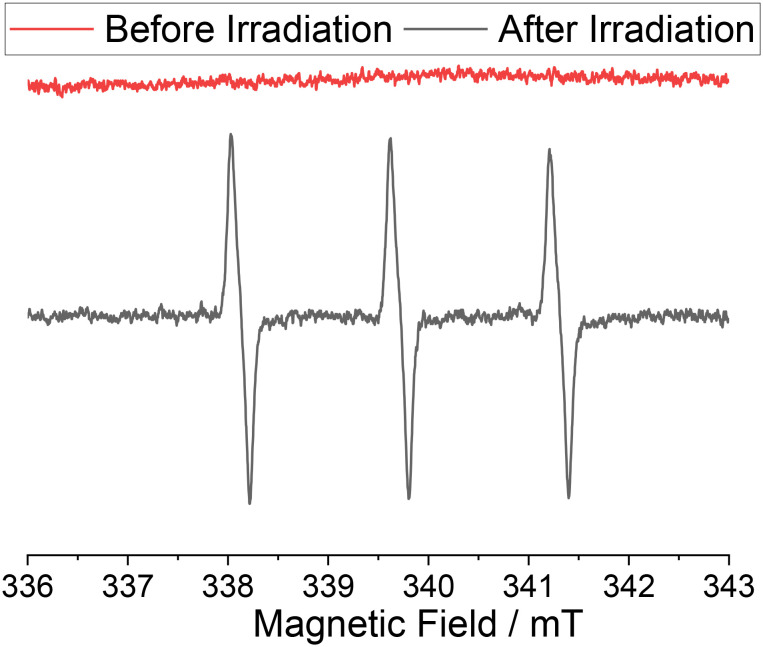
EPR spectra of an oxygen-saturated aqueous solution of TEMP before (red) and after (black) irradiation for 30 minutes with a high-power LED (*λ*_max_ = 405 nm).

Fig. 2 shows the corresponding EPR spectra before and after 30 min of continuous irradiation at 405 nm. We employed 405 nm for irradiation, as thioxanthone exhibits significant absorbance at this wavelength, whereas polystyrene does not demonstrate absorbance (see Fig. 1). This ensures that the incident light is predominantly absorbed by the photo-active thioxanthone. Before irradiation, no signals are detected, whereas a characteristic triplet signal, attributable to the persistent TEMPO radical with a nitrogen hyperfine coupling constant of *a*_N_ = 1.596 mT is observed after light exposure. This further supports the formation of photoactive **TX** moieties on the polystyrene backbone and illustrates its potential as a heterogeneous photocatalyst (see also SI, Fig. S9).

### Assessing polystyrene-bound **TX** as a heterogeneous photocatalyst for alcohol oxidation

As a proof-of-concept, we employ **TX@PS** as a heterogeneous photocatalyst for two model reactions: the oxidation of benzyl alcohol (1) to benzaldehyde (2) and the oxidation of hydroxymethylfurfural (5-HMF; 3) to 5-(hydroxymethyl)-5-hydroxyfuran-2(5*H*)-one (4) and formic acid (5). The reactions were chosen to demonstrate the potential of the photocatalyst to work both in organic solvents (*e.g.* acetonitrile: Table 1) and under aqueous conditions (Table 2) and proceed *via* singlet oxygen: upon excitation of **TX@PS** and subsequent energy transfer leading, the singlet oxygen acts as an oxidant either *via* hydrogen atom transfer or direct insertion reaction.^10,13^

**Table 1:** Catalyst screening, reusability, and control experiments for the oxidation of compound 1*via* singlet oxygen generated according to Scheme 2^a^

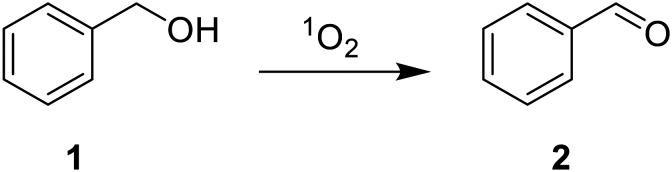	
Modifications of reaction conditions^a^	Product yield^b^ [%]
None	73
Catalyst re-used; second run	60
Catalyst re-used; third run	51
4-hour irradiation	4
No light	1
No catalyst	0
No light, *T* = 70 °C	1

a Reagents and conditions: benzyl alcohol (10 mmol), **TX@PS** (50 mg) in acetonitrile (1 ml) at RT; 405 nm LED irradiation under O_2_ atmosphere for 18 h.

b Determined by ^1^H NMR spectroscopy.

**Table 2:** Catalyst screening, reusability, and control experiments for the oxidation of compound 3*via* singlet oxygen generated according to Scheme 2^a^

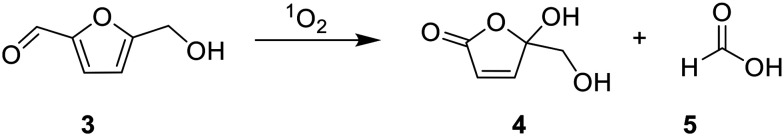		
Modifications of reaction conditions^a^	Conversion^b^ [%]	Product ratio 4 : 5^b^ [%]
None	>99	24 : 76
Catalyst re-used; second run	>99	27 : 73
Catalyst re-used; third run	>99	30 : 70
4-hour irradiation	59	50 : 50
No light	1	—
No catalyst	2	—
No light, *T* = 70 °C	1	—

a Reagents and conditions: hydroxymethylfurfural (10 mmol), **TX@PS** (50 mg) in water (1 ml) at RT; 405 nm LED irradiation under O_2_ for 18 h.

b Determined by ^1^H NMR spectroscopy.

Product yields for the oxidation of benzyl alcohol (1) to benzaldehyde (2) reach 73% in the first run and decrease to 51% upon subsequent reuse of the same catalyst (Table 1). This reduction in catalytic efficiency is potentially due to photobleaching of the chromophore upon prolonged irradiation.^27–29^ Reducing the irradiation time from 18 to 4 h significantly reduces the reaction yield to only 4%. This illustrates the necessity of long-term irradiation. Virtually no formation of 2 was detected without the photocatalyst or when irradiation was omitted, rationalizing the reaction pathway displayed in Scheme 2, where **TX@PS** produces singlet oxygen *via* energy transfer upon irradiation (SI, Fig. S5 and S6). Whilst under our reaction conditions, the solution heated up to a maximum of only 35 °C, it is still important to evaluate the influence of elevated temperature on the catalytic activity.^30^ Therefore, we performed the experiment in the presence of **TX@PS** at elevated temperature (*T* = 70 °C), without light irradiation. Again, no product is detected, underpinning that light irradiation is necessary for catalytic activity.

The oxidation of 5-HMF (3) is well documented and can afford multiple products, such as 5-hydroxymethyl-2-furancarboxylic acid, 2,5-diformylfuran, 2,5-furandicarboxylic acid, 5-(hydroxymethyl)-5-hydroxyfuran-2(5*H*)-one, and formic acid, depending on the reaction conditions.^31–33^ Under our conditions, only 5-(hydroxymethyl)-5-hydroxyfuran-2(5*H*)-one (4) and formic acid (5) were detected as products (SI, Fig. S7). The oxidation of 5-HMF (3) proceeded quantitatively (>99%), even after catalyst reuse. When the irradiation time was shortened from 18 h to 4 h, the conversion of 3 decreased substantially to 59%. In the absence of either the photocatalyst or light, no significant educt conversion was observed, even at elevated temperatures of 70 °C (2%, 1% and 1%, respectively), confirming that **TX@PS** functions as a heterogeneous photocatalyst for singlet oxygen generation (SI, Fig. S7–S9). To determine the relative amounts of 4 and 5 formed, we compared the integrals of characteristic NMR resonances (3.71 ppm (2H) for 4; 8.08 ppm (1H) for 5).^33^ In all three catalytic runs, 5 was the major product; however, the ratio of 4 to 5 shifted upon catalyst reuse (from 24 : 76 in the first run to 30 : 70 in the third; Table 2 and SI, Fig. S7). This trend again suggests changes in the catalytically active species, consistent with our observations for the oxidation of 1 (see above).

The product yields obtained from our model reactions using **TX@PS** as a heterogeneous photocatalyst are comparable to those reported in previous studies:^34–36^ specifically, the oxidation of benzyl alcohol to benzaldehyde achieved a 73% product yield with our heterogeneous system, whereas a comparable homogeneous approach using **TX** in solution yielded up to 82%.^36^ Despite the slightly lower yield, our method offers significant practical advantages, including a simplified work-up process and catalyst reusability, owing to the heterogeneous nature of the **TX@PS** photocatalyst. For the oxidation of 5-HMF, **TX@PS** exhibits high product yields even after repeated reuse of the heterogeneous photocatalyst, consistent with results reported for other organic dyes.^33^

## Conclusions

Herein, we present a proof-of-concept study on the grafting and immobilization of **TX** on **PS**. The material exhibits activity as a heterogeneous photocatalyst for alcohol oxidation *via* a singlet oxygen pathway. For the oxidation of benzyl alcohol in an organic solvent (acetonitrile) and hydroxymethylfurfural in an aqueous solution, product yields of 73% and 99% are achieved, respectively. Tables 3 and 4 show a comparison of **TX@PS** with different photo-catalytic systems employed for the oxidation of benzyl alcohol and 5-HMF, respectively: For the oxidation of benzyl alcohol to benzaldehyde, **TX@PS** achieves product yields of 73%; different systems reported in literature show yields ranging from 36–99% (Table 3). Therefore, our photo-catalyst demonstrates photocatalytic performance comparable to or exceeding that of as established systems reported in literature.

**Table 3:** Comparison of different catalytic systems used in the oxidation of benzyl alcohol

Catalyst	Reaction conditions	Yield/%	Reaction runs	Ref.
**TX@PS**	18 h, 405 nm, CH_3_CN, O_2_, RT	73	3 runs	This work
Bi_4_Ti_3_O_12_ nanosheets	5 h, 360 nm, benzo-trifluoride, atm, RT	36	—	37
BiOBr/g-C_3_N_4_	5 h, Xe lamp, in bulk, atm, RT	36	4 runs	38
Photoactive MOF	16 h, white light EtOH/H_2_O/H_2_O_2_, atm, RT	75	3 runs	39
CsPbBr_3_/Ni_4_P_2_	6 h, >400 nm, in CH_3_CN, O_2_, RT	99	3 runs	40
Nano-TiO_2_	6 h, >365 nm, in CH_3_CN, atm, 30 °C	76	—	41

**Table 4:** Comparison of different catalytic systems used in the oxidation of 5-HMF

Catalyst	Reaction conditions	Yield/%	Reaction runs	Ref.
**TX@PS**	18 h, 405 nm, H_2_O, O_2_, RT	99	3 runs	This work
In–SnS_2_	2 h, 450 nm, CH_3_CN, atm, RT	73^a^	3 runs	42
g-C_3_N_4_/NaNbO_3_	8 h, Xe lamp, H_2_O, O_2_, RT	36^b^	5 runs	43
(Cu_2_O)/TiO_2_	90 min, Xe lamp, H_2_O, O_2_, RT	23^a^	5 runs	44
SGH-TiO_2_	4 h, 515 nm, CH_3_CN, atm, RT	52^a^	5 runs	45

a Main product: 5-formyl-2-furancarboxylic acid.

b Main product: 2,5-diformylfuran.

For the oxidation of 5-HMF, **TX@PS** achieves conversion and product yields of exceeding 99%; different systems reported in literature also show quantitative educt conversion and product yields ranging from 23–73% (Table 4). Importantly, is has to be noted that depending on the catalyst and reaction conditions, different main products (5-formyl-2-furancarboxylic acid or: 2,5-diformylfuran) are reported.

In addition to high product yields, **TX@PS** exhibits chemical stability towards the solvents employed, facilitates a simple work-up procedure and has high reusability. Our findings suggest that the integration of organic dyes with polystyrene provides a straightforward platform for heterogeneous photocatalysis.

## Conflicts of interest

There are no conflicts of interest to declare.

## Supplementary Material

CY-016-D5CY01464F-s001

## Data Availability

The data supporting this article have been included as part of the supplementary information (SI). Supplementary information is available. See DOI: https://doi.org/10.1039/d5cy01464f.
