# Supplementary material for: Polystyrene-bound thioxanthone – a heterogeneous photocatalyst for alcohol oxidation via singlet oxygen production
Source: Catal Sci Technol. 2026 Feb 2;16(5):1790–6. doi: 10.1039/d5cy01464f (PMC12887755; doi:10.1039/d5cy01464f)
Supplement: CY-016-D5CY01464F-s001 [file CY-016-D5CY01464F-s001.pdf]

Supplementary Information (SI) for Catalysis Science & Technology.
This journal is © The Royal Society of Chemistry 2026

# Supporting Information

# Polystyrene-Bound Thioxanthone – A Heterogenous Photocatalyst for Alcohol Oxidation via Singlet Oxygen Production

Max Schmallegger,*[a] Luis Herbst,[a,b] Renata Raptova,[a] Mathias Wiech,[a] Dana Dvoranova,[c] Dmytro Neshchadin,[a] Michal Zalibera[c]

## Contents

*Photos of Reference Polystyrene Systems* 2

*ATR-IR Spectra* 3

*$^1$H NMR Spectra* 4

*EPR Spectra* 8

*Gas Sorption Measurements* 9

*References* 10

## Photos of Reference Polystyrene Systems

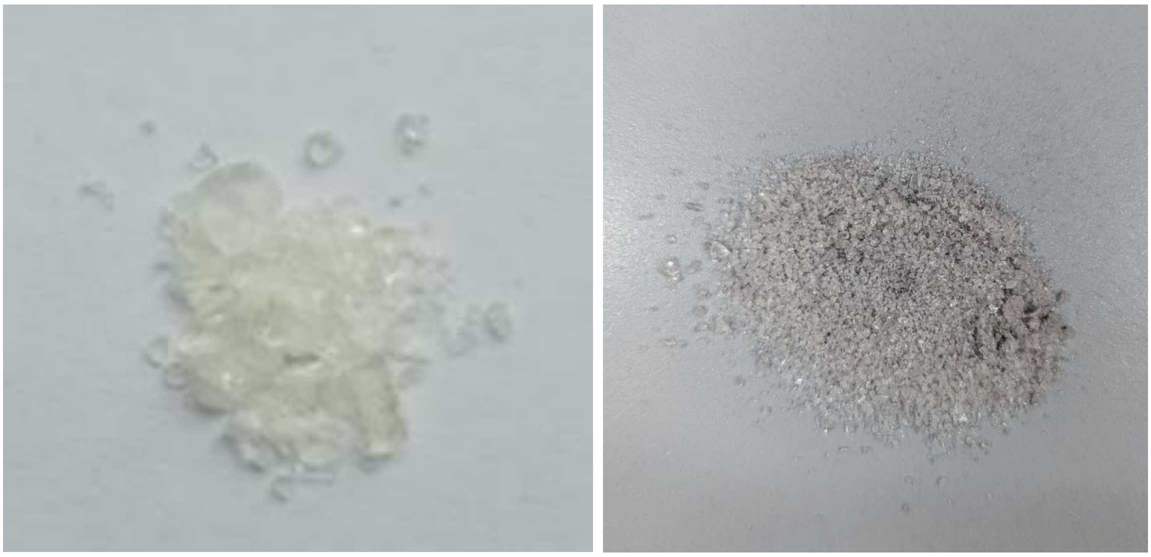

**Scheme S1.** Photograph of polystyrene (left) and polystyrene heated in sulfuric acid at 65° C for 3 hours (right) rationalizing that the colour change in **TX@PS** is due to the formation of covalently bound thioxanthone moieties

## ATR-IR Spectra

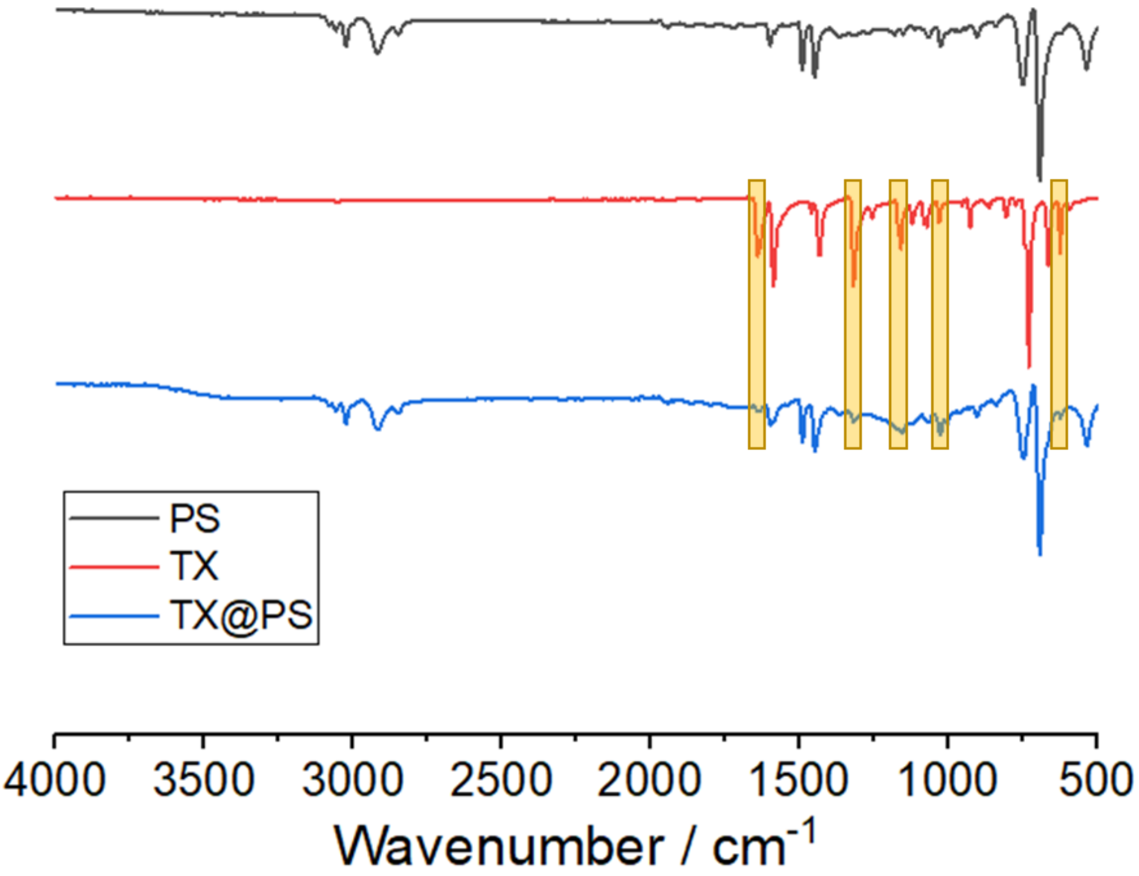


**Figure S1.** FT-IR of **PS** (black), **TX** (red) and **TX@PS**; all spectra were recorded in solid state in reflectance mode – yellow lines indicate characteristic vibrations corresponding to **TX** rationalizing the formation of photo-active moieties on the PS backbone

## $^1$H NMR Spectra

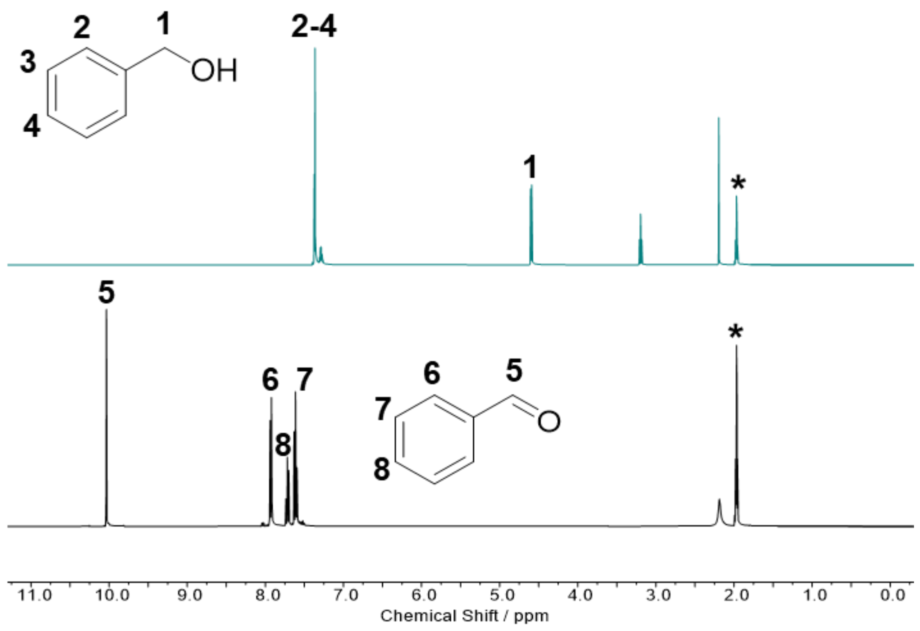


**Figure S2.** Refence $^1$H-NMR spectra and signal assignment of benzyl alcohol (top; signals labelled 1-4) and benzaldehyde (bottom, signals labelled 5 - 8) in $CH_3CN-d_3$; the solvent peak is denoted by an asterisk

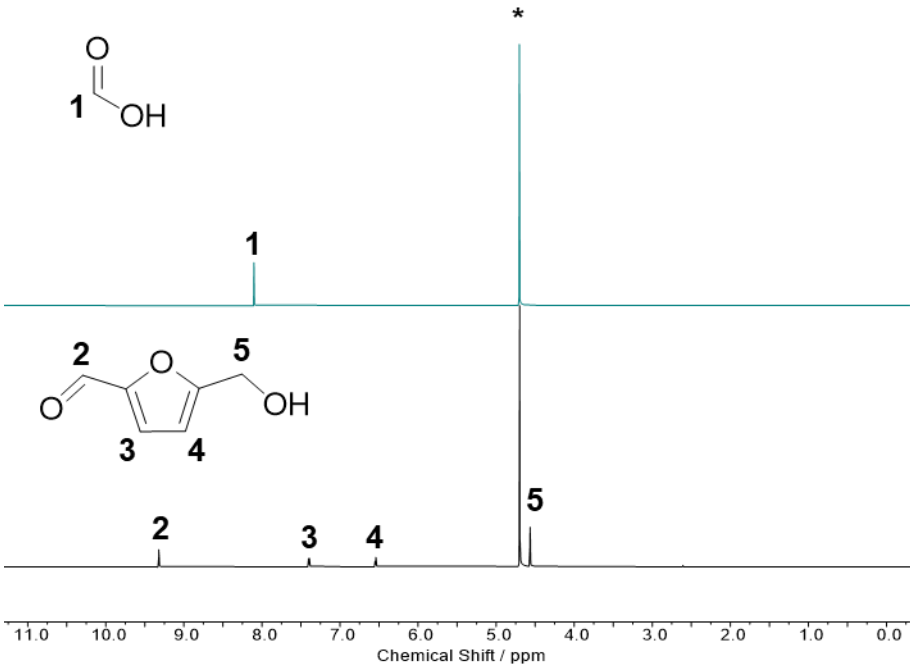


**Figure S3.** Refence $^1$H-NMR spectra and signal assignment of formic acid (top, signal labelled 1) and 5-HMF (bottom, signals labelled 2-5) in $H_2O-d_2$; the solvent peak is denoted by an asterisk

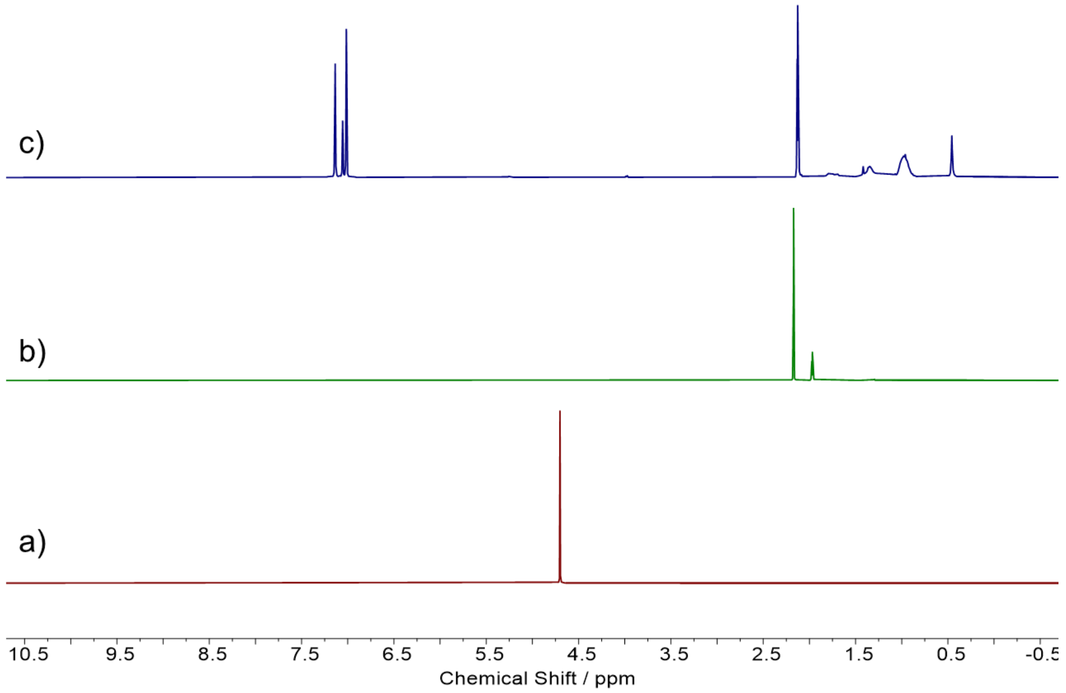


**Figure S4.** $^{1}$H-NMR spectra of **TX@PS** after storage in a) $H_2O$-$d_2$, b) $CH_3CN$-$d_3$ or c) toluene-$d_8$ at 70°C for 24 h; in $H_2O$-d and $CH_3CN$-$d_3$, only solvent peaks are detected, rationalizing the stability of **TX@PS,** whilst in new peaks are detected, indicating that **TX@PS** is not stable under these experimental conditions

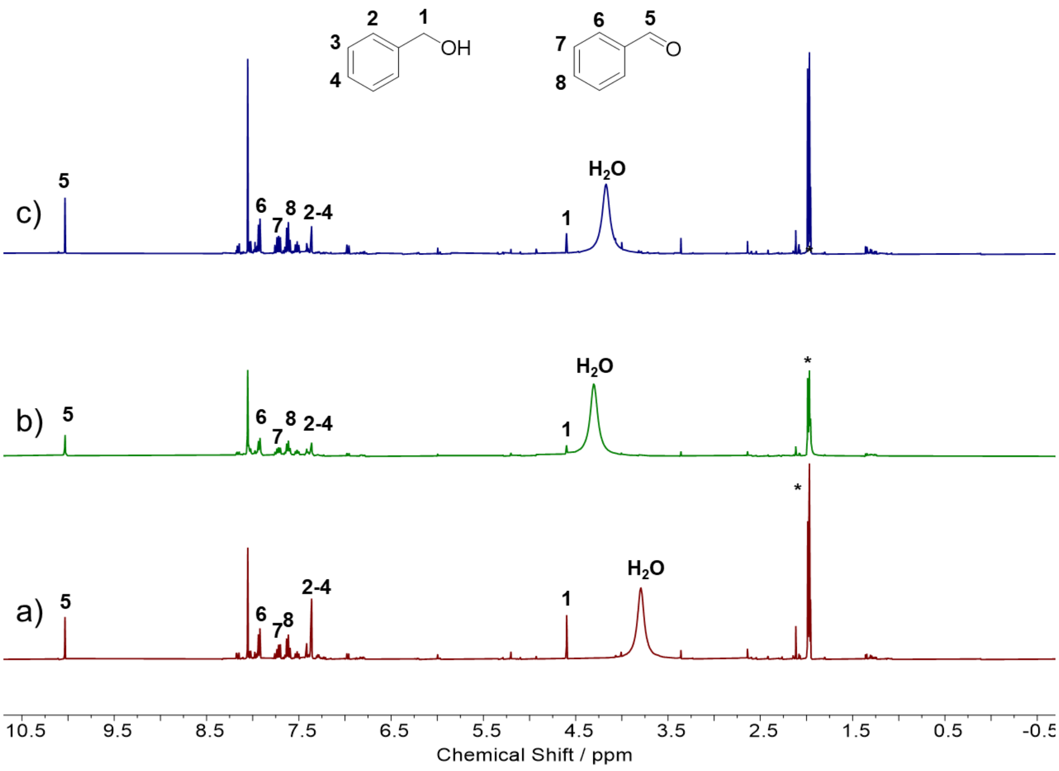


**Figure S5.** $^{1}$H-NMR spectra of **TX@PS** in the presence of benzyl alcohol upon irradiation (18h, 405 nm) with a) fresh catalyst, b) re-used catalyst (once) or c) re-used catalyst (twice); signals labelled 1 – 4 correspond to the educt benzyl alcohol, signals labelled 5 – 8 correspond to the product benzaldehyde; spectra were recorded in $CH_3CN$-$d_3$; the solvent peak is denoted by an asterisk

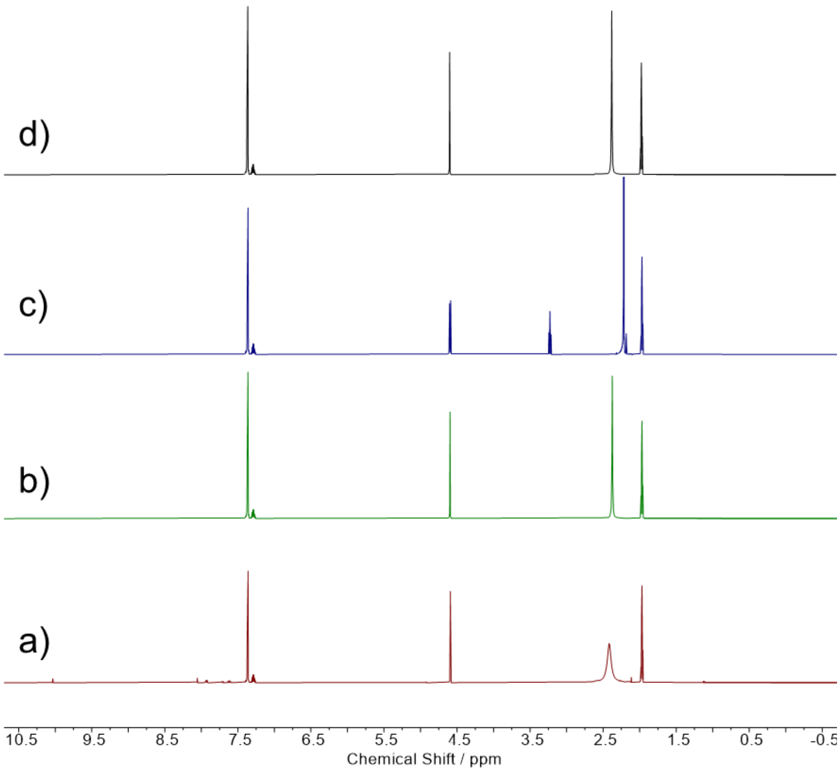


**Figure S6.** Reference $^{1}$H-NMR spectra of benzyl alcohol; a) in the presence of **TX@PS** upon irradiation for 4h; b) in the presence of **TX@PS** without irradiation (18h); c) in the absence of **TX@PS** upon irradiation (18h); d) in the presence of **TX@PS** without irradiation (18h) at elevated temperature (T = 70°C); spectra were recorded in $CH_3CN$-$d_3$

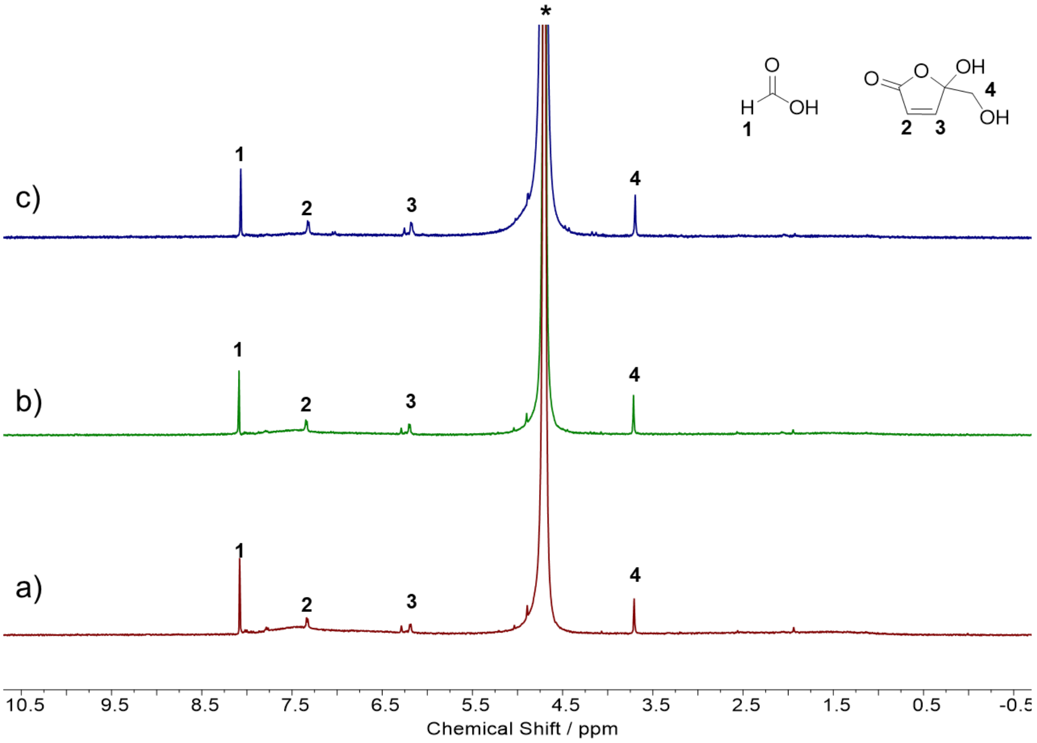


**Figure S7.** $^{1}$H-NMR spectra of **TX@PS** in the presence of **5-HMF** upon irradiation (18h, 405 nm) with a) fresh catalyst, b) re-used catalyst (once) or c) re-used catalyst (twice); the signals labelled 1 correspond to the product formic acid, signals labelled 1 - 4 correspond to the product 5-(hydroxymethyl)-5-hydroxyfuran-2(5H)-one[1]; no signals corresponding to the educt **5-HMF** are detected, indicating full conversion; spectra were recorded in $H_2O$-$d_2$; the solvent peak is denoted by an asterisk

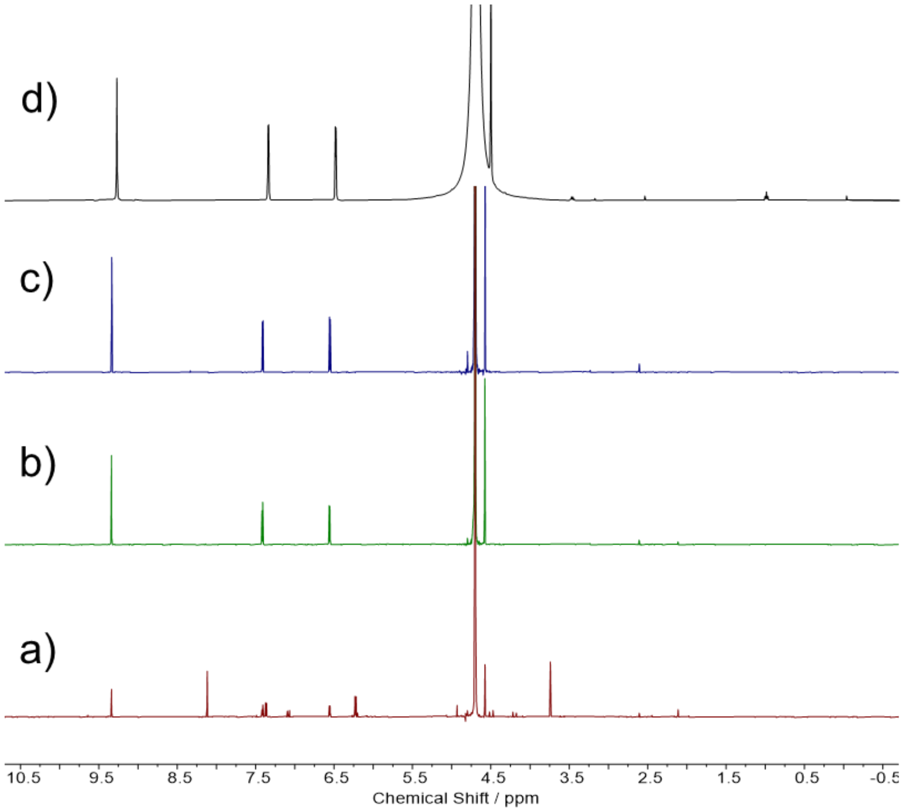


**Figure S8.** Reference $^{1}$H-NMR spectra of 5-HMF; a) in the presence of **TX@PS** upon irradiation for 4h; b) in the presence of **TX@PS** without irradiation (18h); c) in the absence of **TX@PS** upon irradiation (18h); d) in the presence of **TX@PS** without irradiation (18h) at elevated temperature (T = 70° C); spectra were recorded in $D_2O$-$d_2$

**EPR Spectra**

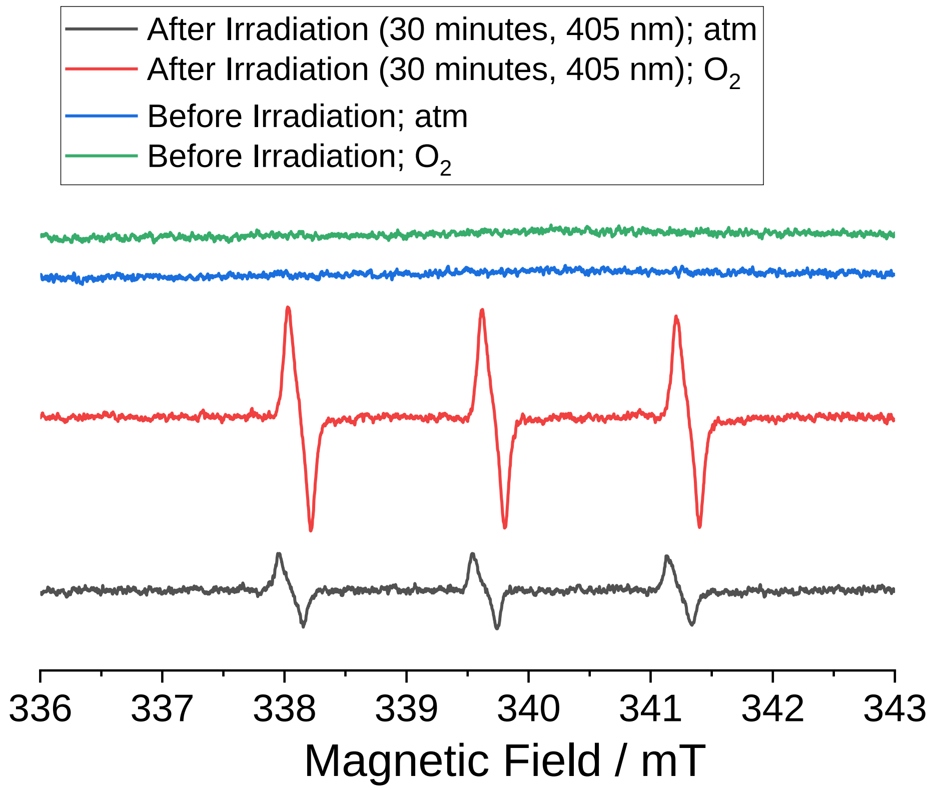


**Figure S9.** EPR spectra of **TX@PS** in the presence of TEMP before and after irradiation for 30 minutes inside a photoreactor ($\lambda_{max}$ = 405 nm) and under atmospheric conditions or under $O_2$ atmosphere; the appearance of signals only after irradiation rationalizes the mechanism discussed in the main text

## Gas Sorption Measurements

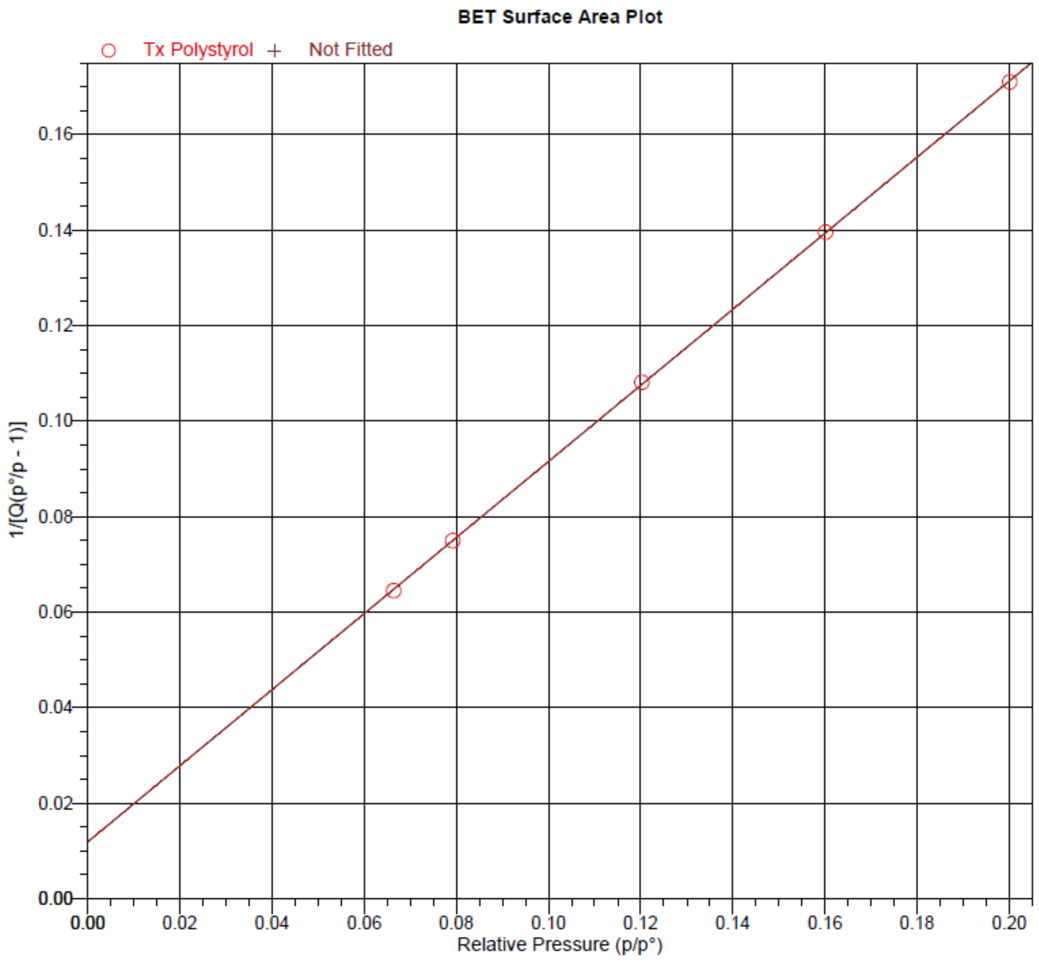


**Figure S10.** Brunauer-Emmett-Teller (BET) analysis of freshly prepared **TX@PS**

## References

**1** T. S. A. Heugebaert, C. V. Stevens and C. O. Kappe, *ChemSusChem*, 2015, 8, 1648–1651.
